# Memory inflation following adenoviral vaccination depends on IL-21

**DOI:** 10.1016/j.vaccine.2018.09.061

**Published:** 2018-11-12

**Authors:** Claire L. Gordon, Claire L. Hutchings, Andrew J. Highton, Julia M. Colston, Nicholas M. Provine, Paul Klenerman

**Affiliations:** Peter Medawar Building for Pathogen Research, University of Oxford, Oxford OX2 3SY, UK

**Keywords:** Adenovirus vectors, Memory inflation, Memory T cells

## Abstract

Cytomegalovirus (CMV) and non-replicating adenoviral vectors can induce expanded, sustained effector-memory CD8^+^ T-cell responses, termed “memory inflation”. During murine CMV (MCMV) infection, CD4^+^ Tcells maintain inflationary virus-specific CD8^+^ T-cell responses via IL-2 but not IL-21. Adenovirus vector vaccination can induce phenotypically, functionally and transcriptionally similar inflationary responses, but it is not known how IL-21 influences the inflating memory response to adenoviral vaccination. Here, we show that IL-21 is an absolute requirement for induction and maintenance of vaccine-derived inflationary CD8^+^ T-cell responses. These data indicate that the induction pathway of inflationary Ad-LacZ T-cells is distinct from inflationary MCMV-specific T-cells and is highly dependent on IL-21. Our observations highlight a fundamental difference in the mechanism by which adenovirus vectors and MCMV drive inflationary T-cell responses.

## 1 Introduction

The success of novel T-cell vaccine strategies depends on the ability of the vaccine to elicit large numbers of long-lived effector memory CD8^+^ T-cells. Cytomegalovirus (CMV) and non-replicating adenoviral vectors can induce an expanded, sustained effector-memory CD8^+^ T-cell response to specific epitopes, termed “memory inflation”, which develop in parallel with conventional non-inflating (contracting) central memory responses to many epitopes [1]. Inflationary T-cells maintain effector function, ability to proliferate and lack features of T-cell exhaustion [2], leading to interest in these cells in vaccine development. Since adenoviral vectors form the basis of many novel vaccines in infectious diseases and cancer this has high translational potential [3].

The recombinant replication-deficient human adenovirus 5 (HuAd5) vector expressing β-galactosidase (Ad-lacZ) induces two distinct pathways for memory – a typical contracting response to one epitope (I8V) and an inflating response to a second epitope (D8V), which is analogous to conventional (M45) and inflating (M38) responses to MCMV infection [4], [5]. While inflationary responses induced by MCMV and Ad-lacZ are phenotypically and functionally similar, the requirement of CD4^+^ T-cell help for the induction and maintenance of T-cell memory differs between MCMV infection and adenovirus vectors. Adenovirus vectors need CD4^+^ T-cell help for the induction, expansion [4], [6], [7], and contraction of memory CD8^+^ T-cell responses [7], while MCMV only needs CD4^+^ T-cell help for the maintenance of CD8^+^ T-cell responses [8]. The mechanism behind this difference has not been explored.

CD4^+^ T-cells produce IL-2 and IL-21 and maintenance of antigen-specific CD8^+^ T-cells in persistent viral infections (i.e. LCMV, HIV-1) has been shown to depend on these cytokines [9], [10], [11]. Studies of MCMV infection have indicated that IL-2 but not IL-21 maintains inflating virus-specific CD8^+^ T-cell responses [8]. In comparison, studies have indicated that non-inflating adenovirus-derived CD8^+^ T-cell memory responses are dependent on CD4^+^ T-cell help, likely through the production of IL-2 and IL-21 [12], [13], however, the dependence of adenovirus-derived inflating memory responses on IL-21 has not been examined.

Here, we show that IL-21 is absolutely required for vaccine-derived inflationary CD8^+^ T-cell responses using a mouse model of Ad-lacZ intravenous immunization. Our observations highlight a fundamental difference between adenovirus vectors and MCMV, especially around the time of memory induction.

## 2 Materials and methods

### 2.1 Mice

Experiments were performed according to UK Home Office regulations (PPL 30/3293, 30/2744, 30/2414) after approval by the University of Oxford ethical review board. *Il21^−/−^* transgenic knock-in mice (Jean Langhorne, National Institute for Medical Research) and C57BL/6 WT mice (Envigo, UK) were kept under conventional conditions in individually ventilated cages and fed normal chow diet.

### 2.2 MCMV and adenoviral vector

MCMV (Strain Smith; ATCC: VR194; from U.H. Koszinoswki, Max von Pettenkofer Institute) and recombinant adenovirus expressing the β-gal protein under the control of the human CMV promoter (Ad-lacZ) was propagated as previously described [4], [5]. Mice were injected intravenously (i.v.) with Ad-lacZ 1 × 10^9^ pfu and/or MCMV 1 × 10^6^ pfu. Recombinant IL-2 was obtained from eBioscience (Paisley, UK).

### 2.3 Detection and analysis of antigen-specific T-cells

Tetramers and fluorochrome-conjugated antibodies are shown in Table S1 and S2, respectively. MHC class I monomers complexed with M38 (H-2Kb), M45 (H-2Db), β gal D8V (H-2Kb) and β gal I8V (H-2Kb) were tetramerized by addition of streptavidin-PE (BD Bioscience) or streptavidin-APC (Invitrogen). Whole blood (100 μl) was stained using a 50 μl tetramer solution at 37 °C for 20 min followed by antibody staining. Viable leukocytes were analyzed using a BD LSR II flow cytometer and FlowJo (Treestar).

### 2.4 Statistical analysis

Statistical analysis was performed using GraphPad PRISM (La Jolla, USA). P-values were determined by two-tailed T test and corrected using Holm-Sidak for multiple comparisons.

## 3 Results

**Fig. 1:**
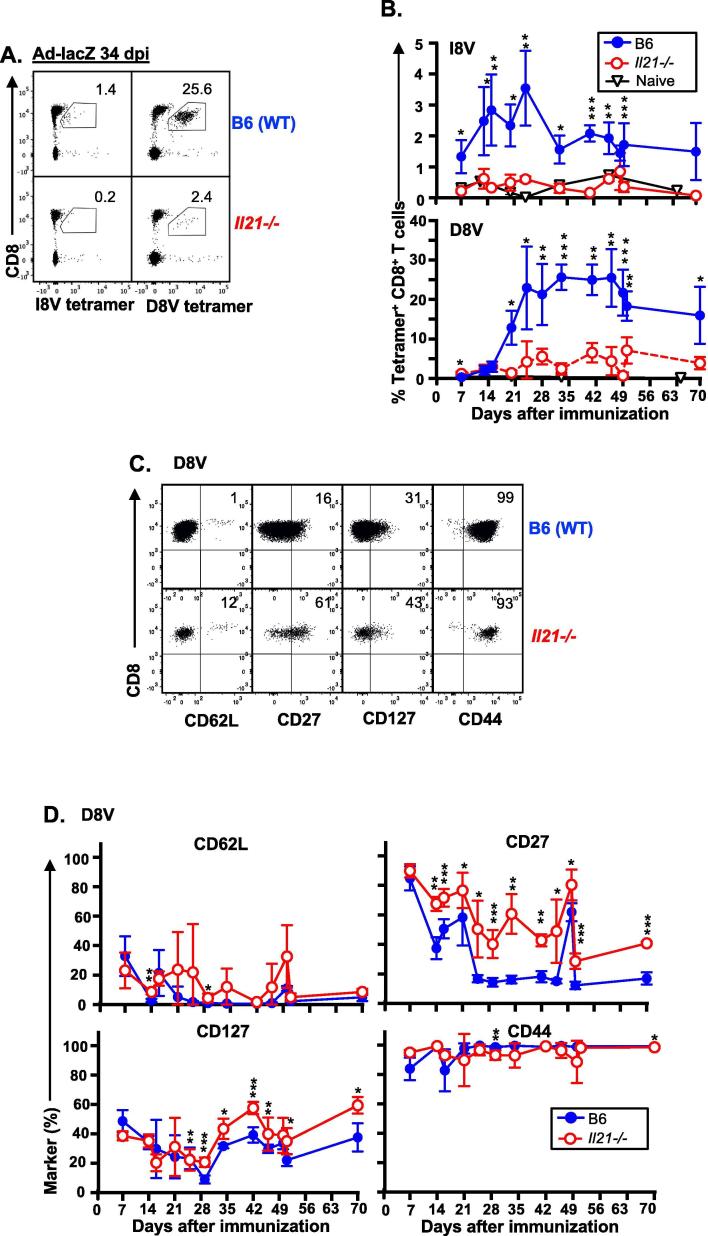
IL-21 is required for conventional and inflating memory responses after Ad-lacZ immunization C57BL/6 (B6, wild type) and *Il21^−/−^* mice were immunized i.v. with Ad-lacZ 1 × 10^9^ pfu and blood serially sampled. Conventional memory responses were assessed by I8V-tetramer staining, and inflationary memory responses were assessed by D8V-tetramer staining. The lower limit of detection of the tetramers is indicated by tetramer staining of naïve B6 and *Il21^−/−^* mice (“Naïve”, black open triangle, n = 3–4 mice) (A) Composite FACS plots (n = 3) of I8V (left) – and D8V (right) -tetramer staining of live lymphocytes in B6 (top) and *Il21^−/−^* mice (bottom) 34 dpi. Mean tetramer^+^ CD8^+^ T-cells are indicated (n = 3). (B) Mean (±SD) I8V (top)- and D8V (bottom)-tetramer^+^ CD8^+^ T-cells (n = 4–5 mice (C) Mean (±SD) I8V (top)- and D8V (bottom)-tetramer^+^ CD8^+^ T-cells (n = 3–5 mice). (D and E) Expression of surface markers CD62L, CD27, CD127 and CD44 on D8V-tetramer^+^ CD8^+^ T-cells. (D) Composite FACS plots (n = 3) of CD62L, CD27, CD127 and CD44 expression on D8V-tetramer^+^ CD8^+^ T-cells in B6 (top) and *Il21^−/−^* mice (bottom) 34 dpi, with numbers indicating mean expression (n = 3). (E) Mean (±SD) expression of CD62L, CD27, CD127 and CD44 on D8V-tetramer^+^ CD8^+^ T-cells in B6 (top) and *Il21^−/−^* mice (bottom) (n = 3–5). Significant differences were determined by T test and corrected for multiple comparisons (Holm-Sidak). P = 0.05 to 0.011 (*), p = 0.01 to 0. 001 (**), p < 0.001 (***). Data is compiled from 5 independent time course experiments (3–5 mice per time point).

Studies indicate that IL-2 but not IL-21 is required for MCMV-induced memory inflation [8]. This is in contrast to non-conventional memory responses to adenoviral vaccination [12], and to other situations of persistent antigen presentation such as chronic viral infection, in which CD4^+^ T-cells support CD8^+^ T-cell responses through IL-21 secretion [10], [11], [14]. We assessed whether IL-21 mediates CD4^+^ T-cell support of inflating Ad-lacZ-derived CD8^+^ T-cell responses by immunizing mice lacking the IL-21 protein (*Il21^−/−^*) with Ad-lacZ. Intravenous immunization with replication-deficient Ad-lacZ induces a classical (non-inflating) memory response to one epitope (I8V) and an inflating response to a second epitope (D8V) [4], [5]. We found that conventional T-cell responses were completely abolished, and that inflationary T-cell responses were markedly reduced in *Il21^−/−^*mice (Fig. 1A and B).

We next asked whether tissue homing, activation state or maintenance profile of inflationary Ad-lacZ-specific CD8^+^ T-cells differed between *Il21^−/−^* and WT mice. We examined lymph node homing based on L-selectin (CD62L) expression, differentiation based on CD27 expression, the homeostatic cytokine IL-7 receptor CD127 (IL-7R) and the memory marker CD44. CD27 is upregulated during the first days after T-cell receptor activation and downregulated during T-cell effector differentiation [15]. CD127 expression is downregulated by activated effector T-cells, and on inflationary CD8^+^ T-cells following response to IL-7 [4]. Overall, inflationary Ad-lacZ-specific CD8^+^ T-cells in *Il21^−/−^* mice retained an effector memory phenotype (CD62L^−^, CD44^+^), but were less differentiated than their WT counterparts (CD27^+^, Fig. 1C and D).

**Fig. 2:**
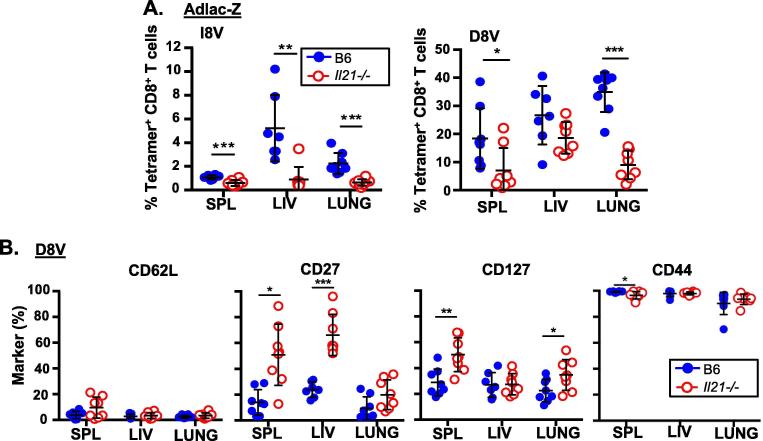
IL-21 is required for tissue CD8^+^ T-cell memory responses after Ad-lacZ immunization (A) Conventional (I8V-tetramer^+^) and inflationary (D8V-tetramer^+^) memory CD8^+^ T-cells responses were assessed in spleen (SPL), liver (LIV) and lung from C57BL/6 (B6 WT, n = 6) and *Il21^−/−^* mice (n = 6) 55–93 days following Ad-lacZ immunization. (B) Expression of surface markers CD62L, CD27, CD127 and CD44 on D8V-tetramer^+^ CD8^+^ T-cells from SPL, LIV and lung from B6 WT and *Il21^−/−^* mice 55–93 dpi. Data is compiled from 2 independent experiments.

We next examined Ad-lacZ-specific T-cell responses in the tissues (spleen, liver and lung) of *Il21^−/−^* and WT mice (Fig. 2). Similar to the blood, conventional memory responses were absent in the tissues of *Il21^−/−^* mice, and inflationary responses in the spleen and lung were significantly reduced (Fig. 2A). Unexpectedly, in contrast to blood and other organs, the magnitude of inflating memory responses in the liver was relatively preserved in *Il21^−/−^* mice although these showed reduced differentiation, as defined by downregulation of CD27 (Fig. 2B). These data suggest that – in contrast to MCMV – optimal induction, maintenance and differentiation of Ad-lacZ-derived inflating memory T-cell responses are dependent on IL-21.

**Fig. 3:**
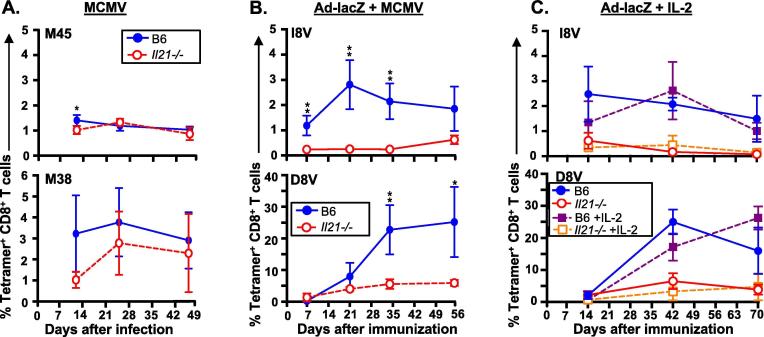
Defective Ad-lacZ-induced inflationary memory responses in *Il21^−/−^* mice cannot be rescued by co-infection with MCMV or IL-2 administration (A) C57BL/6 (B6) and *Il21^−/−^* mice were infected i.v. with MCMV 1 × 10^6^ pfu and blood serially sampled. Mean (±SD) M45 (top)- and M38 (bottom)-tetramer^+^ CD8^+^ T-cells (n = 4 mice per time point) are shown. (B) B6 and *Il21^−/−^* mice were simultaneously infected i.v. with Ad-lacZ and MCMV and blood serially sampled. Mean (±SD) I8V (top)- and D8V (bottom)-tetramer^+^ CD8^+^ T-cells (n = 3–4 mice per time point) are shown. (C) B6 and *Il21^−/−^* mice were immunized i.v. with Ad-lacZ and 2ug IL-2 or PBS was intra-peritoneally injected daily for 5 doses. Blood was serially sampled. Mean (±SD) I8V (top)- and D8V (bottom)-tetramer^+^ CD8^+^ T-cells (n = 3 mice per time point) are shown. Significant differences were determined by T test and corrected for multiple comparisons (Holm-Sidak). P = 0.05 to 0.011 (*), p = 0.01 to 0. 001 (**), p < 0.001 (***).

We investigated whether vaccine-derived inflationary CD8^+^ T-cell responses could be “rescued” from their reliance on IL-21. Because IL-21 is not needed for memory inflation following MCMV infection [8], we asked whether co-infection with MCMV could provide the necessary factors to overcome the inhibition of adenovirus-vaccine-induced memory inflation observed in *Il21^−/−^* mice. Simultaneous co-infection with MCMV and Ad-lacZ does not impact the magnitude of inflationary responses [5]. First, we infected WT and *Il21^−/−^* mice with 1 × 10^6^ pfu MCMV and confirmed that IL-21 was not required for MCMV-specific inflationary (M38-tetramer^+^) T-cell responses (Fig. 3A). Next, we infected B6 and *Il21^−/−^* mice simultaneously with 1 × 10^6^ pfu MCMV and 1 × 10^9^ pfu Ad-lacZ. We observed that MCMV did not rescue inflationary Ad-lacZ-specific T-cell responses in *Il21^−/−^* mice (Fig. 3B), indicating that the requirement for IL-21 is regulated by the precise microenvironment where CD8^+^ T-cells interact with antigen presenting cells during priming. These data indicate that the induction pathway of inflationary Ad-lacZ-specific CD8^+^ T-cells is distinct from inflationary MCMV-specific cells and is highly dependent on IL-21.

Studies have shown that IL-2, likely derived from CD4^+^ T-cells, is important during CD8^+^ T-cell expansion in response to adenovirus vector vaccination [13] and is needed for MCMV-induced memory inflation [8]. We examined whether IL-2 supplementation could rescue vaccine-derived inflating T-cell responses in *Il21^−/−^* mice (2 μg IL-2 i.p. first 5 days following immunization). Administration of IL-2 did not improve conventional or inflationary adenovirus vaccine-derived T-cell responses (Fig. 3C), although the administration of IL-2 may have been too transient to have a long-term effect. These data suggest that IL-21 and possibly IL-2, each play critical non-redundant roles.

## 4 Discussion

Understanding the induction and maintenance of memory inflation is important for the development of CD8^+^ T-cells vaccines that aim to induce large numbers of memory CD8^+^ T-cells capable of providing protection against complex pathogens and cancers. The requirement of CD4^+^ T-cell help for the induction and maintenance of T-cell memory has been described to differ between MCMV infection and adenovirus vectors. MCMV-induced memory inflation requires CD4^+^ T-cell help for maintenance and is dependent on IL-2 but not IL-21 [8]. In contrast, we found that adenovirus-induced memory inflation is dependent on IL-21 for optimal induction, maintenance and differentiation. These data show that although MCMV infection and Ad-LacZ immunization induce inflationary CD8^+^ T-cell responses that are functionally and phenotypically similar, there are parallel pathways of memory induction with the adenovirus vector-pathway highly dependent on IL-21.

The relevant sources of IL-21 required for memory inflation in the adenovirus vaccine model (as opposed to the MCMV model) were not examined in this study. IL-21 – like IL-2 – can be derived from different cellular sources, including both CD4^+^ and CD8^+^ T cells [16]. In MCMV it has been shown that CD8^+^ T cell-derived autocrine IL-2 production is critical for optimal memory inflation [17]. Further adoptive transfer or bone marrow chimera studies to define the cellular source(s) of IL-21 necessary or sufficient for memory inflation in the adenovirus model would be valuable in this context. Similarly, to what extent exogenous boosting with IL-21 could further augment T cell responses driven by adenovirus vaccines has a clear translational implication. This approach has already been taken using others vectors, with clear benefit in pre-clinical models [18] – but given the important differences seen here between vectors, this type of approach could have specific relevance in the development of adenovirus vectors now widely used in clinical vaccine studies.

The differences in the fundamental biology MCMV infection and Ad-lacZ immunization may account for differing pathways of induction of memory inflation. Ad-lacZ is a persistent replication-incompetent vector while MCMV is a persistent viral infection characterized by sporadic low-level reactivation events. It is thought that the need for CD4^+^ T-cell help during priming occurs in systems where innate immune activation is more modest, as occurs with replication-incompetent vectors [6]. In MCMV infection innate responses are robust enough to drive the CD4^+^-independent expansion of inflationary CD8^+^ T-cells (as occurs in acute LCMV infection [19]), such that CD4^+^ T-cell-derived IL-21 is not needed [8]. In contrast, adenovirus vectors induce comparatively modest and transient innate immune responses and the induction of a robust CD8^+^ T-cell response is more dependent on CD4^+^ T-cell help which may require multiple signals, including IL-21. In addition, while both MCMV infection and Ad-lacZ-derived antigens persist, the nature of persistence is quite different. For adenovirus vectors, there is a set amount of antigen that is maintained at some level and then slowly declines over time [4], [20]. In comparison, during MCMV infection, the virus pool is maintained – reactivation events allow viral spread to new cells with accompanying innate immune activation. We hypothesized that these differences in antigen persistence and innate immune activation relate to the differential role for specific CD4^+^ T-cell pathways in helping CD8^+^ T-cell responses. However, we also show that the use of MCMV as an adjuvant in the absence of IL-21 was not sufficient to drive the induction of adenovirus-specific CD8^+^ T-cell memory inflation. Therefore, taken together our data indicates that the induction pathway of inflationary Ad-lacZ CD8^+^ T-cells is distinct from inflationary MCMV-specific cells and is highly dependent on IL-21.

One surprising observation was the relative preservation of liver-associated inflationary populations (not seen in non-inflationary pools), which showed reduced differentiation (CD27hi). In other settings IL-21 is critical for hepatic immunity (e.g. in response to HBV [21]). We hypothesize that this observation may be potentially linked to the local immune-regulatory features in the liver, for example local survival signals mediated by liver sinusoidal endothelial cells [22].

In conclusion, we demonstrate that IL-21 plays a crucial role in the development of inflationary memory T-cell responses following adenovirus-vector vaccination. These data have implications for novel vaccine strategies aiming to promote large and sustained memory T-cell responses.

## 5 Author contributions

C.H., A.J. H and J.M.C performed the mouse experiments. C.LG, C.H., A.J.H and P.K. analyzed the data. C.L.G., N.M.P. and P.K. wrote the manuscript. All authors attest they meet the ICMJE criteria for authorship.
