# Supplementary material for: Memory inflation following adenoviral vaccination depends on IL-21
Source: Vaccine. 2018 Nov 12;36(46):7011–6. doi: 10.1016/j.vaccine.2018.09.061 (PMC6219444; doi:10.1016/j.vaccine.2018.09.061)
Supplement: Supplementary Data 1 [file mmc1.docx]

**Table S1: List of tetramers used for studies antigen-specific CD8^+^ T cells**

| **HLA type** | **Target-epitope** | **Amino acid sequence** | **Abbreviation** | **Source** |
| --- | --- | --- | --- | --- |
| H-2Kb | βgal_497-504_ | ICPMYARV | I8V | NIH Tetramer Facility |
| H-2Kb | βgal_96-103_ | DAPIYTNV | D8V | NIH Tetramer Facility^a^ |
| H-2Db | MCMV-M45_985-993_ | HGIRNASFI | M45 | NIH Tetramer Facility^a^ |
| H-2Kb | MCMV-M38_316-324_ | SSPPMFRV | M38 | NIH Tetramer Facility^a^ |

^a^Peptide for monomer construction was obtained from Proimmune.

**Table S2: Fluorochrome-conjugated antibodies used in flow cytometry**

| **Antibody** | **Fluorochrome** | **Clone** | **Manufacturer^a^** |
| --- | --- | --- | --- |
| CD8 | eF450 | 53-6.7 | eBioscience |
| CD62L | AF700 | MEL-14 | BioLegend |
| CD44 | FITC | LG.7F9 | eBioscience |
| CD27 | PerCp-Cy5.5 | LG3A.10 | BioLegend |
| CD127 | PE-Cy7 | A7R34 | eBioscience |
| Viability | LIVE/DEAD near IR | NA | Life Technologies |

^a^ Antibodies were obtained from BD Biosciences (Oxford, UK), eBioscience (Loughborough, UK), BioLegend (London, UK) and Life Technologies (Loughborough, UK).

Abbreviations: alexa Fluor (AF), allophycocyanin (APC), brilliant violet (BV), eFluor (eF), infrared (IR), peridinin-chlorophyll-protein (PerCP), phychoerythrin (PE) and fluorescein isothiocyanate (FITC).
